# Progressive increase of brain gray matter volume in individuals with regular soccer training

**DOI:** 10.1038/s41598-024-57501-4

**Published:** 2024-03-25

**Authors:** Ju Li, Yaping Cao, Minghao Huang, Zhe Qin, Jian Lang

**Affiliations:** 1 College of P.E. and Sports, Beijing Normal University, Beijing, 100875 China; 2 College of P.E. and Sports, Northwest Normal University, Gansu, 730070 China

**Keywords:** Soccer, Gray matter, Source-based morphometry, Casual structural covariance network, Neuroscience, Sensorimotor processing, Somatosensory system

## Abstract

The study aimed to investigate alterations in gray matter volume in individuals undergoing regular soccer training, using high-resolution structural data, while also examining the temporal precedence of such structural alterations. Both voxel-based morphometry and source-based morphometry (SBM) methods were employed to analyze volumetric changes in gray matter between the soccer and control groups. Additionally, a causal network of structural covariance (CaSCN) was built using granger causality analysis on brain structural data ordering by training duration. Significant increases in gray matter volume were observed in the cerebellum in the soccer group. Additionally, the results of the SBM analysis revealed significant increases in gray matter volume in the calcarine and thalamus of the soccer group. The analysis of CaSCN demonstrated that the thalamus had a prominent influence on other brain regions in the soccer group, while the calcarine served as a transitional node, and the cerebellum acted as a prominent node that could be easily influenced by other brain regions. In conclusion, our study identified widely affected regions with increased gray matter volume in individuals with regular soccer training. Furthermore, a temporal precedence relationship among these regions was observed.

## Introduction

The learning of motor skills refers to the process of gradually improving the speed and accuracy of executing movements through repeated training. The acquisition of motor skills involves an initial phase of rapid short-term learning, during which performance improves quickly, followed by a long-term phase of slow learning, during which performance improvement is incremental. Motor skill training has a significant impact on brain plasticity and is closely related to the duration of the training period.

In recent decades, some studies have examined the relationship between motor learning and brain structural plasticity. Some of these investigations have relied on cross-sectional approaches. For instance, in a study by Maguire et al.^1^, taxi drivers exhibited a significantly larger gray matter volume in the hippocampus. Similarly, several studies found that musicians displayed increased gray matter volume in the right auditory cortex, sensorimotor cortex, premotor cortex, and cerebellum^2–4^. These findings suggest a potential relationship between gray matter volume and specialization on specific skill. Conversely, other studies employed longitudinal designs, capturing brain structural data at different time points. A seminal study by Draganski et al.^5^ found that after three months of juggling practice, MT/V5 and the posterior intraparietal sulcus exhibited increased activation, followed by a return to baseline levels without practice. Furthermore, Driemeyer observed that just 7 days of juggling training could result in increased gray matter volume in MT/V5^6^. Marco et al.^7^ reported a significant increase in gray matter volume in the bilateral supplementary motor area, left superior frontal gyrus, right middle frontal gyrus, and left supramarginal gyrus as a result of balance training and identified frontal and parietal brain areas displayed transient and slowly evolving structural changing patterns. These findings suggest that motor learning induces temporal dynamic alterations in gray matter within activity-dependent brain regions. Dayan proposed that short-term motor skill training primarily involves a cortical network specific to learned movements. In contrast, long-term motor skill training engages a bihemispheric cortical–subcortical network^8^.

In comparison to studies that predominantly concentrate on motor sequence learning (juggling), sports may exert distinct influences on brain structural plasticity, as they involve a combination of diverse cognitive and motor skills, setting them apart from the more focused nature of motor sequence learning. Athletes, with their intensive, specialized training from a young age, provide insights into the effects of complex motor learning on brain plasticity. Research on college basketball players by Park et al.^9^ found that basketball players demonstrated bigger gray matter volume in the vermian lobules VI–VII (declive, folium, and tuber). Additionally, Jancke et al.^10^ found professional golfer had significant bigger gray matter volume in premotor cortex than novices. These findings suggest that individuals undergoing long-term professional sporting train or those possessing high-level specific sports skills exhibit a correlation with larger gray matter volumes in brain regions encompassed by the sensorimotor, visual cortex, and cerebellum.

However, Cross-sectional studies inherently offer only transient insights into changes in gray matter, a limitation that becomes particularly evident in sports training, where results often persist for decades. The complexity and time-consuming nature of implementing such studies further compound these limitations. Furthermore, the intricacy of professional sports training, which extends beyond simpler activities like juggling or balance training, challenges the adequacy of univariate analysis methods. Specifically, techniques like Voxel-Based Morphometry (VBM) may not sufficiently elucidate the brain structural alterations associated with sports training. This complexity necessitates a more nuanced approach to capture the multifaceted impact of professional sports training on brain structure. Recently, a multivariate, data-driven approach proves to be more effective in delineating the structural characteristics of the brain across a broad spatial scale^11^. Furthermore, Zhang et al.^12^ introduced Granger causality analysis (GCA) as an effective method for characterizing temporal changes in gray matter volume, based on a cross-sectional study. Traditionally, Granger causality analysis (GCA) has been a staple in the analysis of functional time-series data. Its application, however, extends beyond this traditional realm. Notably, GCA has been effectively applied to morphometric data, particularly when ordered by progression markers like disease duration. This methodological extension has yielded significant insights, especially in characterizing the flow of brain information in various neurological conditions. For instance, in temporal lobe epilepsy, schizophrenia, Parkinson’s disease, and Alzheimer’s disease, GCA has demonstrated its utility in elucidating complex brain dynamics^13–15^. These applications underscore the versatility and robustness of GCA in diverse research contexts.

Our study builds upon these methodologies, employing VBM for initial comparisons of gray matter volume between regular soccer trainees and a control group. We then apply a multivariate approach, investigating discrepancies in gray matter networks. Finally, we utilize GCA to explore temporal changes in gray matter volume across different durations of soccer training. Our hypothesis posits that differences in brain gray matter volume will primarily manifest in the cerebellum, visual, and sensorimotor cortex. We anticipate that the multivariate approach will more sensitively detect gray matter changes associated with soccer training compared to VBM, and that GCA will reveal the evolving nature of brain structural changes throughout the course of soccer training.

## Subjects and method

### Subjects

A total of 77 participants were recruited for the study, including 36 individuals in the soccer group and 41 individuals in the control group (Table 1). All participants in the soccer group were selected from the college and major in soccer. They underwent soccer training at least three times a week, with each session lasting two and a half hours throughout their professional careers. The total training period ranged from 1 to 15 years. The control group consisted of age-matched regular college students who had never been involved in soccer activities. All participants were healthy, right-handed, had normal vision, had no history of mental illness, and met the criteria for MRI scanning. All participants provided informed consent before the experiment. The experimental protocol received approval from the Institutional Review Board of the Key Laboratory of College of Physical Exercise and Sport at Beijing Normal University, in compliance with the ethical guidelines of the 2013 version of the Helsinki Declaration.

**Table 1:** Demographic and information of subjects.

	Soccer	Control	P
BMI	20.7 ± 1.7	21.4 ± 1.9	0.08
Age	19.3 ± 1.3	19.5 ± 1.6	0.7
Gender	Male: Female: (18:18)	Male: Female:(24:17)	0.45

### MRI acquisition

All the subjects in this study were scanned with a Siemens 3.0T MRI scanner (Trio Tim, Erlangen, Germany) using an eight-channel head coil, including high-resolution T1 weighted imaging (T1WI). The parameters were as follows: T1WI using a Magnetization Prepared Rapid Gradient Echo (MPRAGE) sequence, TR/TE = 2250/2.6 ms, slice thickness = 1 mm, flip angle = 9°, FOV = 256 × 256 mm^2^, and matrix size = 256 × 256.

### T1 preprocessing

The present study utilized high-resolution T1-weighted imaging data, which underwent preprocessing with the CAT12 (Computational Anatomy Toolbox) toolbox of the SPM12 software package in order to eliminate motion and other artifacts. CAT12 is considered to be more accurate than VBM8 for analysis of volume changes^16^. All participants' images were segmented into white matter, gray matter, and cerebrospinal fluid. Subsequently, the images were normalized to the Montreal Neurological Institute (MNI) standard space, and modulated using the Diffeomorphic Anatomical Registration Through Exponential Lie Algebra (DARTEL) toolbox^17^. After assessing the quality using CAT12 to eliminate any inconsistent images, eight subjects were excluded (four belong to soccer group). Finally, the rest gray matter images were smoothed with a Gaussian kernel of 8 mm full-width at half maximum (FWHM).

### Voxel-based morphometry

Using SPM12 software, two-sample t-tests were performed on the preprocessed GM images to compare the differences in GMV between the soccer and control groups. Subsequently, to explore the relationship between training period and morphological changes, which might reveal the possible impact of training period on GMV. Age, gender and total incranial volume were regressed as covariates. P < 0.05 was considered to be statistically significant and family-wise error (FWE) correction with an extent threshold of 20 contiguous voxels was used.

### Source-based morphometry

In this study, the SBM analysis was conducted using the GIFT toolbox (https://trendscenter.org/trends/software/gift). Firstly, the gray matter images of all subjects were arranged to form an m × n gray matter matrix (G), where m is the total number of voxels and n is the number of subjects. A neural network Infomax algorithm was employed to perform Independent Component Analysis (ICA) on the gray matter matrix, decomposing it into a mixing matrix (M) and a source matrix (S). The size of the mixing matrix was k x n, where k represents the number of components. Each column indicates how each component is expressed in every participant, referred to as loading scores. These scores convey the contribution of each component to the 77 subjects in the study. Based on previous studies that used the minimum description length (MDL) principle and had similar sample sizes, the k was determined to be 10^18,19^. The source matrix represents the relationship between different components and each voxel, and its size is m × k. Each row represents the contribution of the component to different voxels, and each column represents the contribution of the voxel to different components. To ensure the reliability of the components, ICA was repeated 100 times using ICASSO (http://research.ics.aalto.fi/ica/icasso/), and the minimum and maximum cluster sizes were set at 80 and 100 respectively.

### Casual structural covariance network

Specifically, if the present value of Y could be predicted more accurately by the combination of the past values of X and Y than the independent past value of Y, X has Granger causal effects on Y.

In this study, all the loading score with significant intergroup difference of soccer group were arranged from shortest to longest training duration, working as ‘pseudo-time series’ data to characterize the progressive alteration of gray matter related to soccer training^12^. Then, we performed the coefficient-based GCA using the Brain Covariance Connectivity Toolkit (BCCT) (https://github.com/JLhos-fmri/BrainCovarianceConnectToolkitV2.1)^20^. GC values were t-transformed to assess statistical differences (P < 0 0.05).

### Statistical analysis

Statistical analyses for this study were conducted using SPSS. Prior to conducting group difference tests for the loading scores, we assessed the normality of the data (SFigure-1, STable-1). If the data met the assumption of normality, we employed a two-sample t-test. For non-normal data, a Mann–Whitney U test was utilized. Furthermore, we conducted a correlational analysis to investigate the relationship between loading scores and training period, while controlling for age and gender as covariates. The significance level for correlational analysis was established at 0.05, and we employed false discovery rate (FDR) correction.

## Results

### Increasing grey matter volume

**Figure 1:**
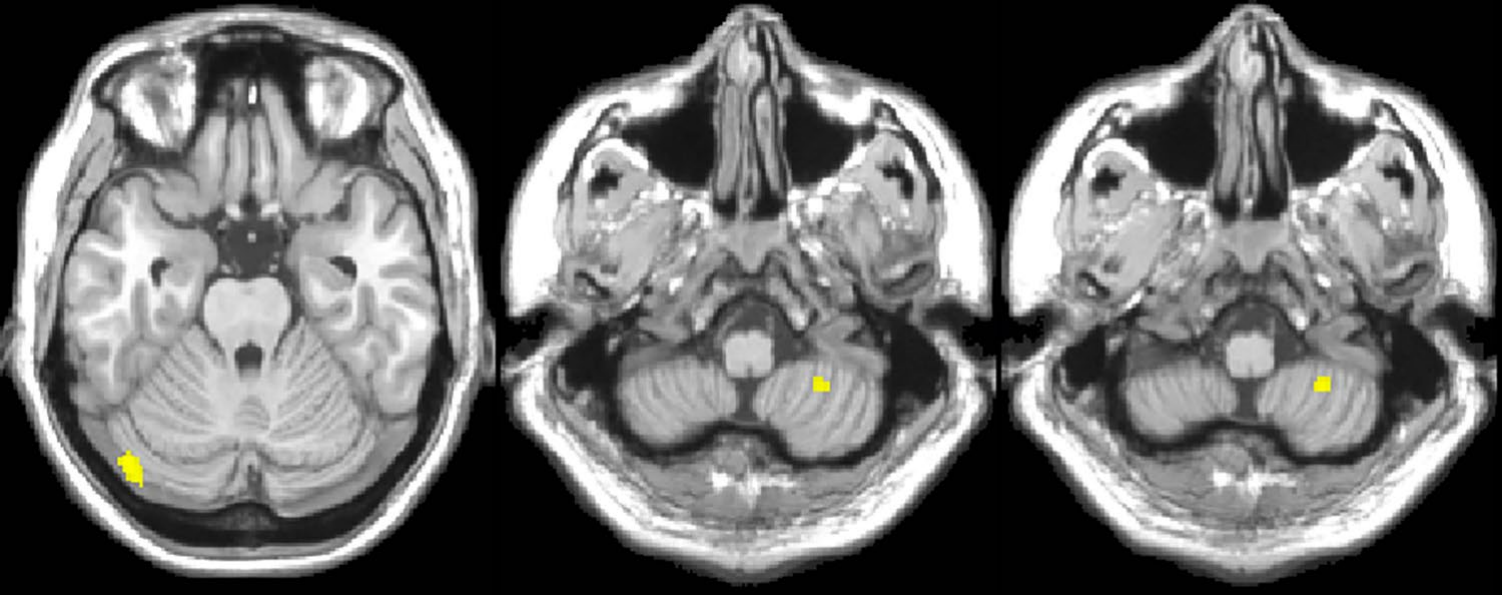
Group comparison of gray matter between soccer and control group using VBM method. The yellow highlights represent regions with larger gray matter volume in the soccer group compared to the control group(FWE < 0.05, Cluster size > 20).

Both VBM and SBM analyses revealed that the soccer group exhibited larger gray matter volumes compared to the control group. VBM results demonstrated soccer group has larger gray matter volume in the right cerebellum_Crus8 and left cerebellum_Crus1 compared to control group(FWE < 0.05) (Fig. 1, Table 2).

**Table 2:** The VBM method identified brain regions with significantly larger gray matter volume in the soccer group compared to the control group (FWE < 0.05, Cluster size > 20).

Brain region	Coordination(MIN)	Voxel	Intensity
Right cerebellum_Crus8	27 − 50 − 57	40	5.6
Left cerebellum_Crus1	− 44 − 78 − 24	53	5.7

**Figure 2:**
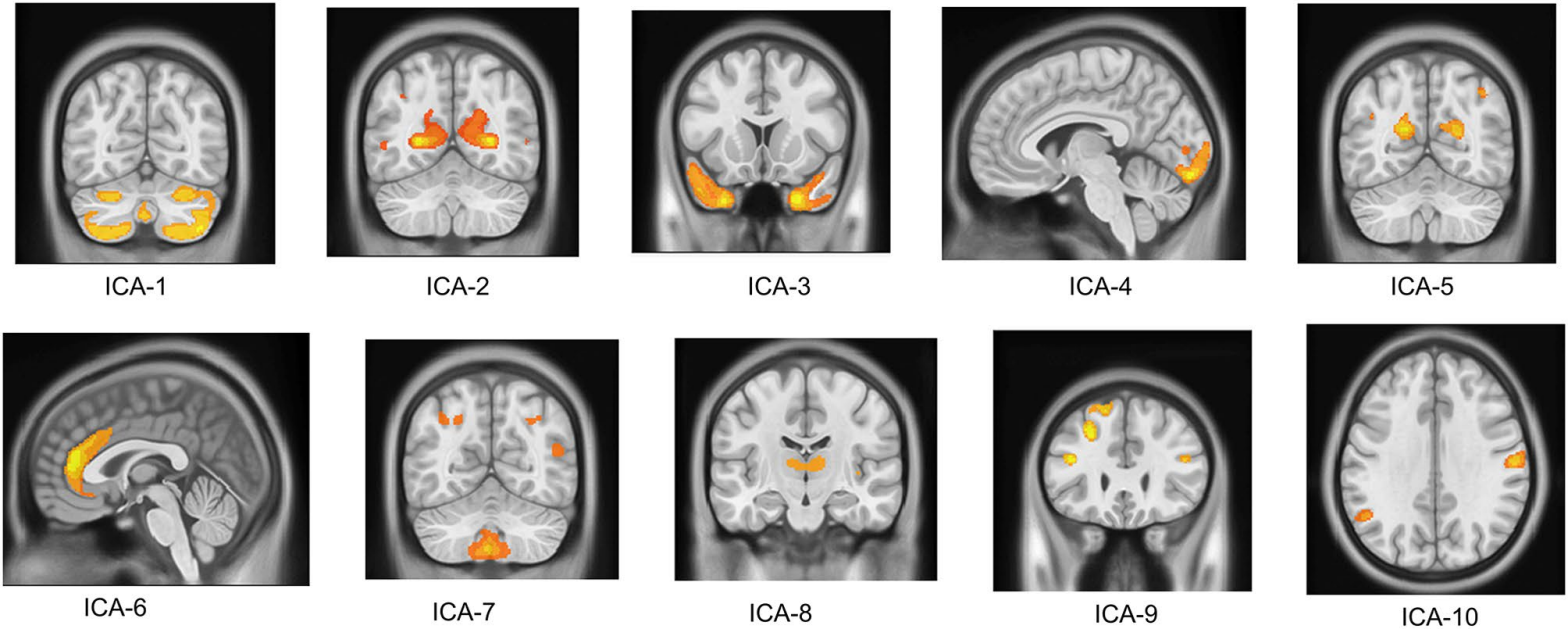
The spatial maps of 10 independent components based on the SBM method.

In the SBM analysis, ten independent components were eventually extracted and labeled 1 to 10. The specific brain regions of each component are shown in detail in Fig. 2 and Table 3.

**Table 3:** The specific locations of brain regions of 10 independent components.

Component	Main brain regions
ICA-1	Bilateral cerebellum_Crus2/4/5
ICA-2	Bilateral calcarine
ICA-3	Bilateral middle temporal pole
ICA-4	Left calcarine
ICA-5	Bilateral cuneus
ICA-6	Right anterior cingulum
ICA-7	Left Cerebellum_Crus9
ICA-8	Bilateral thalamus
ICA-9	Left superior/middle frontal lobe
ICA-10	Left Angular, Right supramarginal

**Figure 3:**
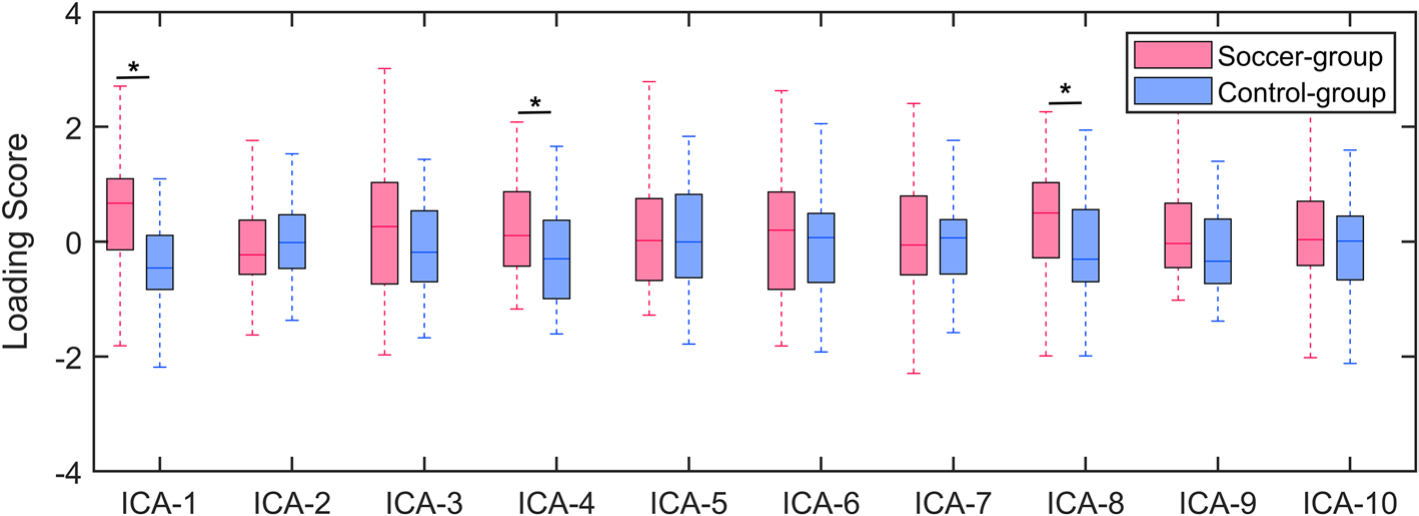
Comparison of loading scores between the soccer and control groups. The star indicates that P < 0.05 with FDR correction.

The between-group comparison of loading score showed that there were three components with significant differences between soccer and control group (P < 0.05 with FDR corrected, Fig. 3, STable-2). In summary, the increased gray matter volume mainly encompassed the cerebellum, calcarine and thalamus.

### Correlation analyses

**Figure 4:**
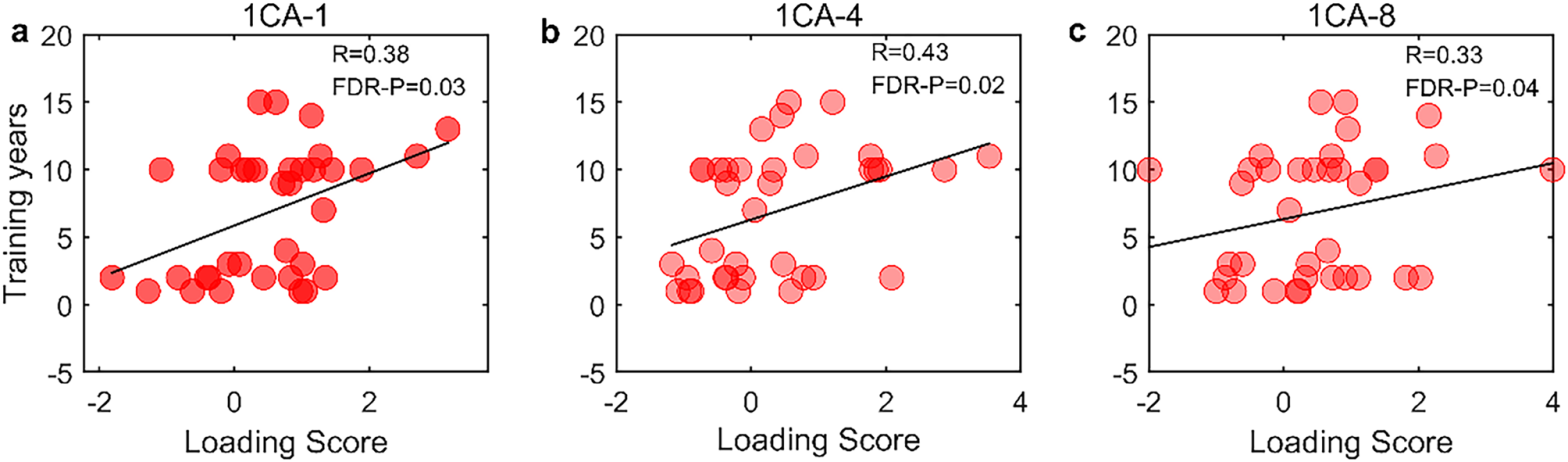
The correlation analysis between loading scores and training period.

The correlational analyses showed that loading score of ICA-1, ICA-4, and ICA-8 had a significant positive correlation with training period (P < 0.05, with FDR corrected, Fig. 4). Furthermore, the gray matter volume of VBM had significantly positive relationship with loading scores of ICA-1, ICA-4 and ICA-8 (P < 0.05, with FDR corrected, SFigure-2).

### Progressive structural increase

**Figure 5:**
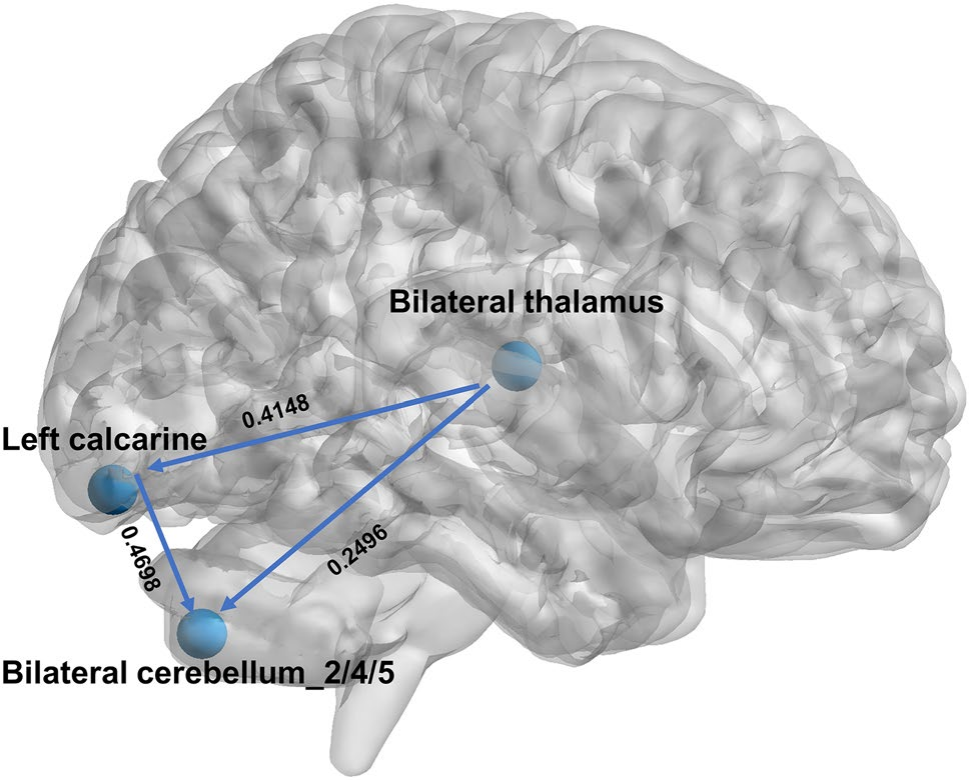
The ROI-based analysis of causal networks of structural covariance.

The ROI-based casual structural covariance network (CaSNC) results revealed that the thalamus exhibited prominent nodes exerting causal effects on other brain regions (Fig. 5, Table 4). The cerebellum region emerged as the prominent node that was most susceptible to the influence of other regions, while the calcarine potentially served as an intermediary node in this network (Fig. 5, Table 4).

**Table 4:** The GC value of granger causal effect between ROI.

Component pair	GC value	p value
Bilateral thalamus (ICA-8) to Left calcarine (ICA-4)	0.4148	0.0007
Bilateral thalamus (ICA-8) to Bilateral Cerebellum_2/4/5 (ICA-1)	0.2946	0.006
Left calcarine (ICA-4) to Bilateral Cerebellum_2/4/5 (ICA-1)	0.4698	0.01

## Discussion

This study employed both VBM and SBM to investigate gray matter characteristics in the soccer population. The results revealed significant increases in the volume of the cerebellum, thalamus, and calcarine cortex. Then, we further constructed a CaSCN to explore the temporal priority relationship among these gray matter increasing regions.

This study represents the first instance of employing both univariate and multivariate approaches to investigate the impact of soccer training on gray matter morphological changes. Notably, there were disparities in the size and intensity of the regions identified by SBM and VBM. Nevertheless, it is important to highlight that nearly all the regions identified by the VBM approach were also detected by SBM. One key advantage of SBM is its multivariate nature, allowing it to consider interrelationships between voxels, thus facilitating the identification of naturally grouped regions. The sources unveiled by SBM provide valuable insights into the localization of gray matter changes. In contrast, VBM, although a powerful and user-friendly approach, does not yield information about the interrelationships among identified regions. SBM exhibited heightened sensitivity, enabling a more comprehensive detection of gray matter morphological changes^21^. On the other hand, VBM displayed greater specificity in pinpointing the most prominent brain regions with deformations^22^. Consequently, the integration of these two methodologies leads to a more comprehensive understanding of the relationship between gray matter morphological changes and soccer training.

In line with the previous research, the most significant gray matter morphological changes were observed in the cerebellum. Specifically, significant differences were observed in left cerebellum_Crus8, right cerebellum_Crus1 and bilateral cerebellum_Crus2/4/5. Additionally, correlation analysis indicated a significant positive correlation between cerebellar volume and years of training. Numerous studies consistently demonstrate that expert athletes in various sports have significantly larger cerebellar gray matter volume compared to non-athletes^9,23–25^. Animal studies also showed that glial volume Purkinje cell, volume of the molecular layer, number of synapses, and dendrite size of stellate cells in the cerebellum were significantly larger in acrobat-trained rats compared to rats engaged in a sedentary lifestyle^26–28^. These findings suggest that physical training can significantly modify the structural plasticity of the cerebellum. The left cerebellum_8 and bilateral cerebellum_4/5 are essential components of the cerebellar motor module, playing a crucial role in controlling motor-related functions such as balance, posture, and motor learning^29^. These brain regions are of particular significance in the context of soccer, as they serve as the foundation for players to execute complex technical movements with precision and skill. Soccer requires saccadic eye movements for tracking and intercepting the ball in motion. Recent fMRI studies have shown that the cerebellum_Crus6/7 regions are activated during visually guided rapid eye movements^30^. Additionally, the right cerebellum_Crus1 and bilateral cerebellum_Crus2 are part of the cerebellar cognitive modules and closely linked to executive functions and semantic process. Beyond the motor module of the cerebellum, which primarily responds to sensorimotor information, the ability to comprehend the intention of an action, and even the underlying strategy at a more abstract level, likely depends on the cerebellum’s cognitive module. Hence, we hypothesized that an increase in gray matter in the cerebellum_Crus1/2 could be associated with improved sports decision-making or prediction resulting from long-term soccer training.

SBM results also demonstrated larger gray matter volume in the bilateral thalamus and Calcarine regions, indicating to some extent that SBM is more sensitive in detecting gray matter morphological changes compared to VBM^21,31^. The thalamus plays a crucial role in the brain, receiving neural projections from the cortex, cerebellum, and subcortex^32^. It acts as a key node in both the direct and indirect pathways, facilitating the integration of information from the basal ganglia and transmitting it to the supplementary motor area, leading to the inhibition or activation of corresponding actions^33^. Thus, the thalamus serves as a central relay station in the brain. Previous studies have found that soccer experts exhibit superior response inhibition ability, which is significantly related to thalamic gray matter volume^34^. Therefore, the increasing thalamic gray matter volume may indicate more coordinated functioning of various action control circuit, facilitating the execution of soccer techniques and tactics.

The calcarine region, located in the posterior part of the occipital lobe, plays a crucial role in visual spatial processing^35^. Previous resting-state brain imaging studies have found that soccer experts exhibit significantly greater recruitment capacity in the visual network compared to novices. Studies have indicated that the calcarine region is part of the action observation network (AON), playing a vital role in action learning and prediction processes^36,37^. However, in terms of structural research, there have been few studies demonstrating a significant increase in calcarine gray matter volume among skilled athletes. This phenomenon can be attributed, on one hand, to the constraints of univariate research methods, and on the other hand, to the fact that previous studies have mostly focused on closed-skill sports such as long-distance running, diving, or gymnastics, where athletes do not heavily rely on visual information to adjust their movements. In contrast, soccer, as a complex team sport, takes place in large and populated competition fields, and vision serves as the primary source of information for players to make sporting decisions and predictions. Compared to novices, soccer experts demonstrate higher gaze efficiency^38,39^. Furthermore, a comprehensive review of studies suggests that during predictive tasks, the visual cortex activation is stronger in skilled athletes^36,40^. Hence, the observed increase in calcarine volume may suggest a potential neural basis for the enhanced observational ability seen in soccer players. Nevertheless, these findings necessitate further validation.

Changes in brain structure over time are best evaluated in longitudinal studies. However, results from this work suggest the potential for assessing temporal changes in the structure of the brain by applying granger causality analysis to morphometric data classified according to training duration. The CaSCN revealed that structural changes in one region precede and allow prediction of the change in another region in relationship to the training peroid^15^. The results of CaSCN demonstrated that the thalamus served as the central hub of the GCA network, with thalamic changes potentially causally linked to all other nodes. During the initial phases of soccer training, interregional information transmission among different brain regions is not yet coordinated. As a relay station in the brain, an increase in thalamic volume has the potential to promote information integration across brain regions and facilitate the rapid acquisition of motor skills^41,42^. Consequently, changes in thalamic gray matter volume may manifest earlier than in other brain regions. There are significant positive GC connections between the cerebellum and the calcarine, suggesting that an increase in calcarine gray matter volume occurs prior to cerebellar changes. Soccer involves complex visual information processing, requiring players to quickly perceive and analyze the movement of the ball, teammates, and opponents on the field^39^. This heightened visual demand may lead to early structural adaptations in the primary visual cortex to enhance visual acuity and processing capabilities. The cerebellum plays a crucial role in motor learning and coordination, making it susceptible to adaptations as soccer players develop and refine their skills over time^43^. Longitudinal studies have also indicated that during the process of juggle training, there is an earlier increase in gray matter volume in the visual cortex compared to the motor control regions^44,45^.

This study utilized VBM and SBM methods to depict brain structural characteristics in individuals with regular soccer training. A combination of univariate and multivariate methods was employed to provide comprehensive information. The findings confirmed that soccer training significantly increased gray matter volume in the cerebellum, thalamus, and calcarine. Moreover, the thalamus may play a central role in driving the augmentation of gray matter in other brain regions.

## Limitations

Some limitations should be noted. Firstly, the CaSCN values were calculated based on temporally organized group data, which can suggest the extension of causal influence but cannot directly depict the precise temporal sequence of soccer training for individual participants. Secondly, the nature of CaSCN makes it challenging to derive values for individual subjects, with available data typically limited to group-level analyses. Therefore, it would be beneficial to conduct longitudinal studies that examine individual covariance networks, allowing for a direct quantification of the temporal precedence of structural alterations on a per-subject basis. Thirdly, it is important to acknowledge that the present study lacks behavioral measurements. Consequently, drawing definitive conclusions about the relationship between structural alterations and behavioral outcomes is challenging. Finally, subjects in the Pro group initiated their professional soccer training at various ages, potentially resulting in some heterogeneity.

### Supplementary Information

Supplementary Information.

## Data Availability

The data presented in this study are available on request from the corresponding author.
