# Supplementary material for: Progressive increase of brain gray matter volume in individuals with regular soccer training
Source: Sci Rep. 2024 Mar 25;14:7023. doi: 10.1038/s41598-024-57501-4 (PMC10963784; doi:10.1038/s41598-024-57501-4)
Supplement: Supplementary file 1 — Supplementary Information. [file 41598_2024_57501_MOESM1_ESM.docx]

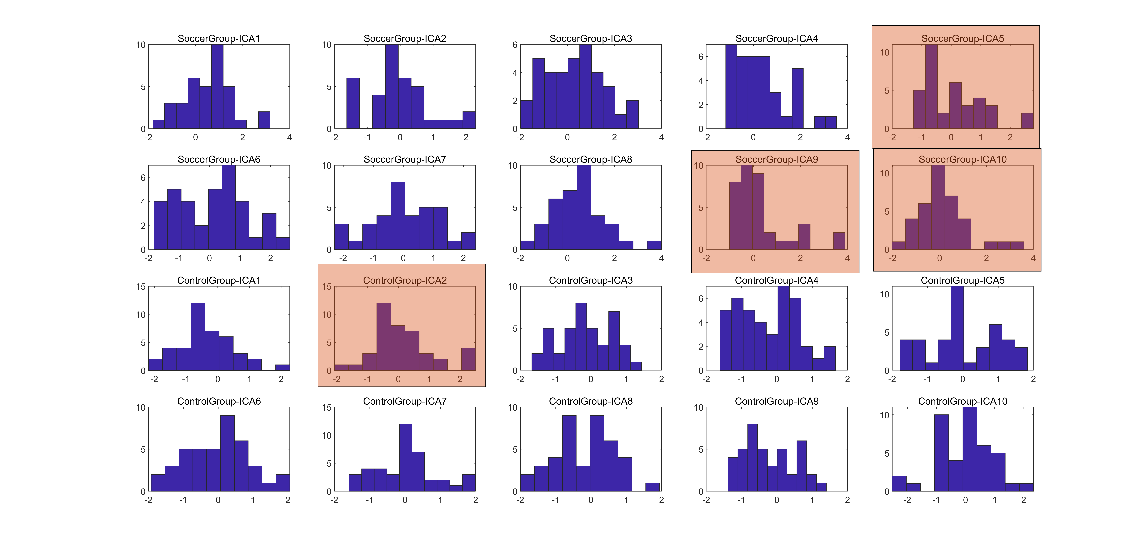


SFigure 1 the histogram of loading scores about the soccer and control group. The red shaded rectangles mean P<0.05, indicating non-normal distribution.

| ICA | Soccer-group | Control-group |
| --- | --- | --- |
| ICA-1 | 0.40 | 0.50 |
| ICA-2 | 0.50 | 0.04 |
| ICA-3 | 0.50 | 0.50 |
| ICA-4 | 0.10 | 0.40 |
| ICA-5 | 0.01 | 0.43 |
| ICA-6 | 0.50 | 0.50 |
| ICA-7 | 0.50 | 0.25 |
| ICA-8 | 0.50 | 0.50 |
| ICA-9 | 0.0010 | 0.08 |
| ICA-10 | 0.0419 | 0.50 |

STable 1 the results of the distribution test on loading scores using the K-S test.

| ICA | P | FDR_P |
| --- | --- | --- |
| ICA-1 | 0.0001 | 0.001 |
| ICA-2 | 0.48 | 0.68 |
| ICA-3 | 0.05 | 0.11 |
| ICA-4 | 0.01 | 0.04 |
| ICA-5 | 0.83 | 0.92 |
| ICA-6 | 0.50 | 0.68 |
| ICA-7 | 0.92 | 0.92 |
| ICA-8 | 0.003 | 0.01 |
| ICA-9 | 0.05 | 0.11 |
| ICA-10 | 0.55 | 0.68 |

STable 2 the results of group comparison in loading scores with FDR correction.


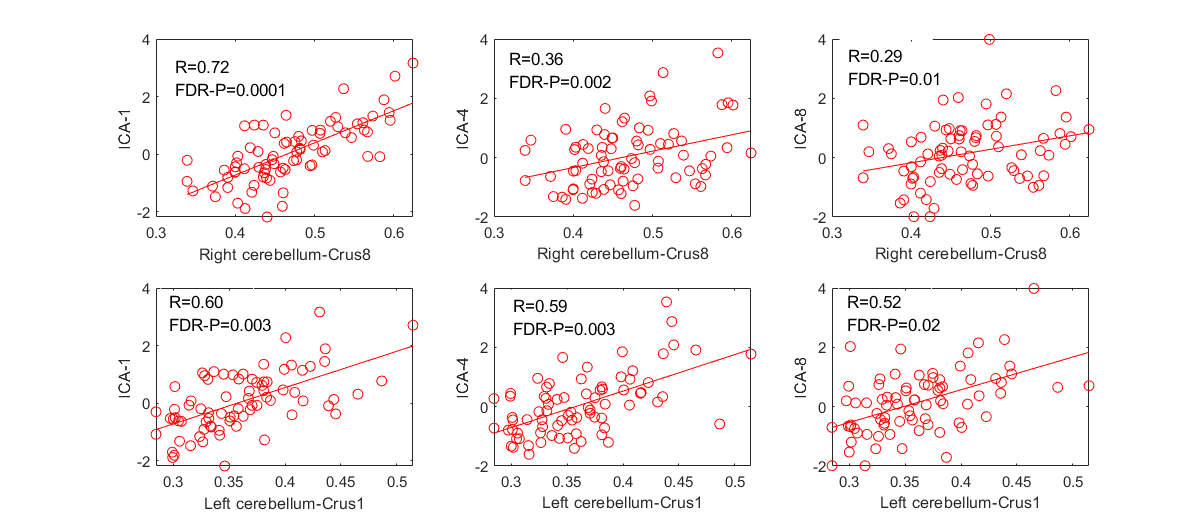


SFigure 2 The correlation analysis between loading scores and Seed density of VBM
